# Comprehensive germline-genomic and clinical profiling in 160 unselected children and adolescents with cancer

**DOI:** 10.1038/s41431-021-00878-x

**Published:** 2021-04-12

**Authors:** Rabea Wagener, Julia Taeubner, Carolin Walter, Layal Yasin, Deya Alzoubi, Christoph Bartenhagen, Andishe Attarbaschi, Carl-Friedrich Classen, Udo Kontny, Julia Hauer, Ute Fischer, Martin Dugas, Michaela Kuhlen, Arndt Borkhardt, Triantafyllia Brozou

**Affiliations:** 1 Department of Pediatric Oncology, Hematology and Clinical Immunology, Medical Faculty, Heinrich Heine University, Düsseldorf, Germany; 2 Institute of Medical Informatics, University of Münster, Münster, Germany; 3 Department of Experimental Pediatric Oncology, University Children’s Hospital of Cologne, Medical Faculty, Cologne, Germany; 4 Pediatric Hematology and Oncology, St. Anna Children’s Hospital, Department of Pediatrics and Adolescent Medicine, Medical University of Vienna, Vienna, Austria; 5 University Children’s and Adolescent’s Hospital, Rostock University Medical Center, Rostock, Germany; 6 Division of Pediatric Hematology, Oncology and Stem Cell Transplantation, Medical Faculty, RWTH Aachen University, Aachen, Germany; 7 National Center for Tumor Diseases (NCT), Dresden, Germany: German Cancer Research Center (DKFZ), Heidelberg, Germany; 8 Present Address: Department of Pediatrics, Pediatric Hematology and Oncology, University Hospital Carl Gustav Carus, Technische Universität Dresden, Dresden, Germany; 9 Present Address: University Children’s Hospital Augsburg, Swabian Children’s Cancer Center, Augsburg, Germany

**Keywords:** Oncogenesis, Paediatric cancer, Cancer genetics

## Abstract

In childhood cancer, the frequency of cancer-associated germline variants and their inheritance patterns are not thoroughly investigated. Moreover, the identification of children carrying a genetic predisposition by clinical means remains challenging. In this single-center study, we performed trio whole-exome sequencing and comprehensive clinical evaluation of a prospectively enrolled cohort of 160 children with cancer and their parents. We identified in 11/160 patients a pathogenic germline variant predisposing to cancer and a further eleven patients carried a prioritized VUS with a strong association to the cancerogenesis of the patient. Through clinical screening, 51 patients (31.3%) were identified as suspicious for an underlying cancer predisposition syndrome (CPS), but only in ten of those patients a pathogenic variant could be identified. In contrast, one patient with a classical CPS and ten patients with prioritized VUS were classified as unremarkable in the clinical work-up. Taken together, a monogenetic causative variant was detected in 13.8% of our patients using WES. Nevertheless, the still unclarified clinical suspicious cases emphasize the need to consider other genetic mechanisms including new target genes, structural variants, or polygenic interactions not previously associated with cancer predisposition.

## Introduction

Cancer is a multifactorial disease associated with genetic as well as non-genetic risk factors. Whereas behavior and lifestyle factors such as sun exposure, infectious diseases, and alcohol abuse are major contributors to cancer development in adults, these factors are negligible in children. Instead inherited and de novo germline variants contribute to about 10% of childhood cancers [1, 2]. One of the first comprehensive germline studies showed that 8.5% of childhood cancers were caused by an underlying known cancer predisposition syndrome (CPS) [1]. Notably, the frequency of detected predisposing germline variants varied between the different tumor entities. The highest incidence was found in non-central nervous system (CNS) solid tumors (16.7%), followed by CNS tumors (8.6%), whereas the lowest prevalence was detected in leukemia (~4.4%). Other studies reported comparable frequencies of about 7–14% of childhood and adolescent cancer patients carrying pathogenic germline variants [1, 3]. These frequencies, although quite similar, might be influenced by the workflow applied to identify pathogenic variants, as well as by the recruitment of the patients, since some studies preselected the patients based on their clinical outcome or clinical signs for CPS [3] or included only patients with a certain tumor type [4].

However, one of the main questions is how to identify the children with genetic cancer predisposition in the clinic, as this has direct implications for clinical decisions and personalized treatment options. Different guidelines have been published for facilitating the identification of patients at risk for having a CPS. These are based on family history, age at presentation, type of cancer, or concomitant clinical findings of skin- or congenital anomalies [5, 6]. However, to date, only one study has applied these criteria to an unselected cohort in order to analyze how well they correlate with genetic findings in cancer predisposition genes (CPGs) [7].

Here, we report on a new prospective study of an unselected patient cohort of 160 children with cancer on which we performed on parent–child trios whole-exome sequencing (WES). The comprehensive medical data of each patient were correlated with the whole-exome sequencing results identifying monogenetic germline variants associated with cancer predisposition in 22/160 patients.

## Methods

### Enrollment of an unselected patient cohort

As described in Brozou et al. [8], we offered all children (<19 yrs) with a newly diagnosed cancer, who were treated at the Department of Pediatric Oncology, Hematology and Clinical Immunology at the University Children’s Hospital since 2015 and their parents, to participate in this study. Four cases were sent by external collaborators due to suspicious clinical features or family anamnesis. Overall, 254 patients were eligible for the study, of which 229 (90.2%) gave positive consent to participate in the study (Supplementary Fig. 1). Of 160 of those, we had sufficient study material (peripheral blood) from the child as well as the parents, which were included in this study. For patients with leukemia, we obtained, if possible, skin fibroblasts which were used for analysis of the germline variants. These patients were not selected based on family history or features suggestive of a cancer-predisposing syndrome. Hence, our study describes an unselected patient cohort. In all cases, informed consent was obtained after at least two consultations with a pediatric oncologist who was trained in genetic counselling. The procedure, aims and benefits of the study, as well as its limitations were explained and discussed with the patient and parents or legal representatives. The study team expressed no recommendations, thereby guaranteeing autonomous decision-making. All patients were made clearly aware that they would be able to retract their consent at any time. The families were given at least one week to consider before agreeing to participate in the study. Once consent was given, we systematically collected demographic and medical data as described [8]. Detailed personal and clinical history was recorded using a published questionnaire tool [5, 6], which requests data including signs of underlying medical conditions and congenital anomalies, as well as a three-generation pedigree regarding malignancies. The study was approved by the ethics committee of Heinrich Heine University, Düsseldorf, Germany (ethics vote number 4886 R and study registration number 2014112933).

### Whole-exome sequencing

We performed whole-exome sequencing on 158 trios, consisting of the patient and the respective parents. For two cases (Case-159 and Case-160), it was not necessary to perform trio whole-exome sequencing as the genetic alterations predisposing to the tumorigenesis were identified during routine workup based on their prominent clinical features (Supplementary Table 1). DNA was extracted from the peripheral blood mononuclear cells of the patients and parents, or, in cases of patients with leukemia, from fibroblasts (if available) using the DNeasy Blood & Tissue kit (Qiagen). Next-generation WES was performed using the SureSelect Human All Exon V5+UTR kit (Agilent). The library was paired-end sequenced on an Illumina HiSeq2500 (2 × 100 bp) or NextSeq550 (2 × 150 bp) sequencer to yield an average on target coverage of ≥80×. Refer to the Supplementary Methods for a detailed description of the bioinformatic processing of the WES data.

All pathogenic, likely pathogenic variants and prioritized variants of unknown signficance were validated using PCR-based Sanger sequencing.

### Interpretation of genetic variants

Evaluation and interpretation of genetic findings can be very challenging and was therefore performed by an interdisciplinary team including basic researchers and senior physicians of the department of Pediatric Oncology, Hematology, and Clinical Immunology. We applied two different published tools for automated variant interpretation, which are based on the ACMG standards and guidelines [9]. The first annotation was based on rules adapted from the CharGer tool [10, 11] as outlined in Supplementary Table 4. The second tool was the pre-published CPSR pipeline [12] to which we uploaded our custom gene lists consisting of 295 genes of category 1–3 (refer to Supplementary Table 2 and the Supplementary Methods for a detailed description of the gene list). Both tools classified the variants as pathogenic (P), likely pathogenic (LP), benign, likely benign, or variant of unknown significance (VUS). According to the ACMG standards [9], the term “pathogenic” applied in our study defines whether a variant in a gene may be pathogenic for the cancer development of the child. Next, we performed a manual review of all variants classified by CharGer and/or CPSR. Using this approach, we also thoroughly examined homozygous and compound heterozygous variants in genes with autosomal recessive inheritance. The manual review included confirmation of the patient´s phenotype being within the spectrum of the associated syndrome or cancer, and a literature review to confirm the pathogenicity of a gene for cancer. Hence, a gene identified as P/LP by the interpretation tools could be manually revised as a VUS. For the classification of variants identified as VUSs by the interpretation tools, we manually reviewed published data on the variant and combined that information with the clinical data of the patient. When we found strong evidence of a variant being associated with the cancer predisposition in that patient, we defined those variants as prioritized VUSs. We uploaded P/LP variants as well as prioritized VUS identified in this study to the ClinVar database (https://www.ncbi.nlm.nih.gov/clinvar/).

## Results

### Description of the unselected patients’ cohort

In order to understand the role and inheritance patterns of genetic tumor predisposition, we recruited 160 children and adolescents (<19 years at first disease onset) diagnosed with cancer and their families. Following informed consent, WES analysis was performed on parent–child trios. We have previously reported on family acceptance to trio-sequencing studies [8] and on single cases [13–16] of this study. A detailed clinical description of the complete cohort of patients is summarized in Supplementary Table 1. The median age of the patients was 4.4 (range 0–18.5) years at disease onset, with a male to female ratio of 1.6:1. Notably, we included one pair of siblings in the study (Case-77, Case-78).

Leukemia, including 48 acute lymphoblastic leukemia (ALL) and 11 acute myeloid leukemia (AML), represented the largest number of cancers included within our cohort (36.9%) (Table 1). 20.0% of the patients harbored a brain tumor (including medulloblastoma (*n* = 9), glioblastoma, or pilocystic astrocytoma (both *n* = 6)). 15.6% of the patients developed a solid tumor with rhabdomyosarcoma being the most common disease in this group (12/25 solid tumors). 14.4% of the patients were diagnosed with a lymphoma (Hodgkin (*n* = 11) or non-Hodgkin lymphoma (*n* = 12)). Non-CNS embryonal tumors represented the smallest fraction of tumor entities (13.1%) including, among others, 11 patients with neuroblastoma. As an unselected large cohort, the frequency of the different tumor entities in our study reflects the distribution of malignancies in the general pediatric population. We performed whole-exome sequencing of 158/160 trios included in this study. For Case-159 (Ataxia telangiectasia) and Case-160 (Beckwith Wiedemann Syndrome), the genetic alterations predisposing for tumorigenesis were already identified during routine workup based on their prominent clinical features, which were indicative of a CPS (Table 2, Supplementary Table 1).

**Table 1:** Overview of patients’ characteristics of pediatric cancer types studied in the unselected cohort of 160 patients.

	Number of cases (Frequency)
Sex	
Male	98 (61.2%)
Female	62 (38.8%)
Age at diagnosis	
0–5 yrs	97 (60.6%)
6–10 yrs	28 (17.5%)
11–15 yrs	28 (17.5%)
16–18 yrs	7 (4.4%)
Diagnosis	
Leukemia	59 (36.9%)
B-cell acute lymphoblastic leukemia	39
T-cell acute lymphoblastic leukemia	9
Acute myeloid leukemia	11
Brain Tumor	32 (20.0%)
Medulloblastoma	9
Pilocytic astrocytoma	6
Glioblastoma	6
Ependymoma	2
Congential brain tumor	2
Primitive neuroectodermal tumor	1
Plexuscarcinoma	1
Glioma	1
Ganglioma	1
Embryonal tumor with multilayered rosettes	1
Atypical teratoid/rhabdoid tumor	1
Astrocytoma	1
Solid tumor	25 (15.6%)
Rhabdomyosarcoma	12
Ewing Sarcoma	6
Osteosarcoma	2
Germcell tumor	2
Clear cell sarcoma of the kidney	2
Epithelioid sarcoma	1
Lymphoma	23 (14.4%)
Hodgkin lymphoma	11
Burkitt lymphoma	4
T-cell lymphoma	3
Diffuse large B-cell lymphoma	3
Anaplastic large cell lymphoma	2
Non-CNS embryonal tumor	21 (13.1%)
Neuroblastoma	11
Nephroblastoma	7
Hepatoblastoma	3

*yrs* years

**Table 2:** Overview of pathogenic/likely pathogenic variants associated with cancer predisposition identified in the patient cohort.

Case; Diagnosis	Gene/ transcript	Chromosomal position in bp (hg19)	Nucleotide change; Amino acid change	Zygosity	Inheritance	Clinical signs	Associated syndrome
Case-68; B-ALL	*PTPN11* ENST00000351677.2	NC_000012.11:g.112915523A>G	c.922A>G; p.(Asn308Asp)	heterozygous	de novo	2	Noonan Syndrome
Case-76; GBM	*MSH6* ENST00000234420.5	NC_000002.11:g.48030698dup	c.3312dup; p.(Gly1105Trpfs*3)	heterozygous	de novo	None	Lynch Syndrome
Case-99; B-ALL	*TP53* ENST00000269305.4	NC_000017.10:g.7578263G>A	c.586C>T; p.(Arg196*)	heterozygous	de novo	3	Li Fraumeni Syndrome
Case-140; RMS	*NF1* ENST00000358273.4	NC_000017.10:g.29665160del	LRG_214t2:c.6819+3del; Splicing	heterozygous	de novo	2	NF type I
Case-7^a^; MB	*MSH6* ENST00000234420.5	NC_000002.11:g.48027547_48027549del	c.2426_2428del; p.(Val809del)	homozygous	Transmitted by mother + father	2	CMMRD
Case-10^b^; RMS	*DICER1* ENST00000526495.1	NC_000014.8:g.95570329dup	c.3405dup; p.(Gly1136Argfs*3)	heterozygous	Transmitted by father	2	DICER syndrome
Case-19; RMS	*TP53* ENST00000269305.4	NC_000017.10:g.7579558_7579596del	LRG_321t1:c.97-6_129del; Splicing	heterozygous	Transmitted by father	2	Li Fraumeni Syndrome
Case-31^c^; Plexus Ca	*TP53* ENST00000269305.4	NC_000017.10:g.7577548C>T	c.733G>A; p.(Gly245Ser)	heterozygous	Transmitted by father	2	Li Fraumeni Syndrome
Case-158; RMS	*TP53* ENST00000269305.4	NC_000017.10:g.7578393A>C	c.537T>G; p.(His179Gln)	heterozygous	Transmitted by father	1	Li Fraumeni Syndrome
Case-159^d^; T-ALL	*ATM* ENST00000278616.4	NC_000011.9:g.108141874G>C	LRG_135t1:c.2921+1G > C; Splicing	homozygous	Transmitted by mother + father	3	Ataxia telangiectasia

*Diagnosis* diagnosis of initial cancer disease, *B-ALL* B-cell acute lymphoblastic leukemia, *Ca* carcinoma, *GBM* glioblastoma, *MB* medulloblastoma, *RMS* rhabdomyosarcoma, *T-ALL* T-cell acute lymphoblastic leukemia, *Clinical signs* number of positive clinical signs indicating a cancer predisposition, *NF type I* neurofibromatosis type I, *CMMRD* constitutional mismatch repair deficiency syndrome

^a^Variant of Case-7 described in detail by Taeubner et al. [15].

^b^Variant of Case-10 described in detail in Fremerey et al. [13].

^c^Variant of Case-31 described in Brozou et al. [8].

^d^*ATM* variant was identified during routine workup in an external diagnostic laboratory.

### Germline variants in cancer predisposition genes

**Fig. 1:**
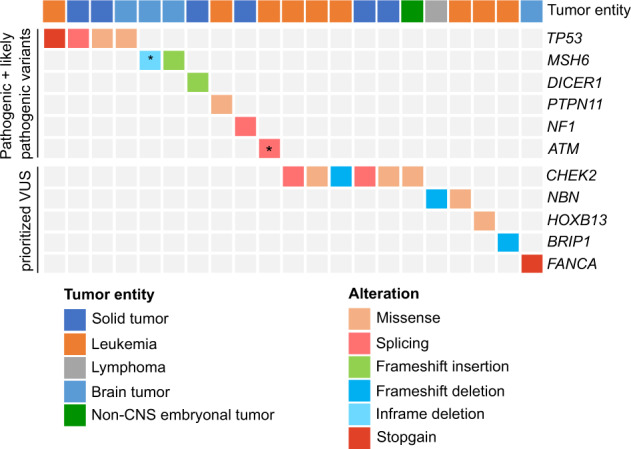
Overview of identified pathogenic/likely pathogenic variants as well as prioritized VUS. We identified 10 pathogenic/likely pathogenic variants in genes associated with cancer predisposition syndromes as well as 11 prioritized VUSs potentially associated with increased cancer risk identified in the unselected cohort of patients. Each line represents a gene and each column represents a case. The top genes harbor pathogenic variants, whereas genes in the lower part carry prioritized VUSs. The color code indicates the type of alteration. Asterisk (*) indicate that the variant has been identified as homozygous in the patient.

In order to identify variants predisposing to cancer, we analyzed 295 genes that we divided into three different categories based on their level of evidence being associated with cancer predisposition. Refer to Supplementary Methods and Supplementary Table 2 for a description of selected genes. We identified 1767 protein-changing variants (frameshift indels, inframe indels, missense, stopgain, startlost, stoploss, consensus splice site, splice region (+/−3–8 bp)) of which 97.9% were transmitted by the respective parents whereas 38 variants were de novo. Applying a standardized bioinformatics workflow for variant interpretation including the automated variant interpreters CharGer [10, 11] and CPSR [12] (Supplementary Methods), we classified after manual revision five variants in category 1 genes as pathogenic (P) and four variants as likely pathogenic (LP) (Supplementary Fig. 2, Supplementary Table 3). In addition, for Case-159 and Case-160, pathogenic variants were identified during routine workup, totaling to 6.9% of analyzed patients carrying a P/LP variant. As none of the variants in the genes of category 2 and 3 were classified as P/LP, we report in the following only on variants detected in genes of category 1. The majority of P/LP variants were present in the group of solid tumors (4/10 variants), whereas three P/LP variants were detected in both patients with brain tumors and leukemia. The most recurrently affected gene was *TP53* (4/10 patients), whereas 2/10 patients harbored P/LP variants in *MSH6* (Fig. 1, Table 2).

Using the following three parameters (i) Charger/CPSR classification, (ii) published data on the variant/gene, and (iii) clinical data of the patient, we identified in eleven patients prioritized VUSs, which we suspect to play a role in cancer predisposition. 6/11 patients carried a prioritized VUS in *CHEK2*, and three of them developed a B-precursor leukemia. 2/11 patients carried prioritized VUSs in *NBN* (Fig. 1, Table 2).

### Clinical signs of cancer predisposition

**Fig. 2:**
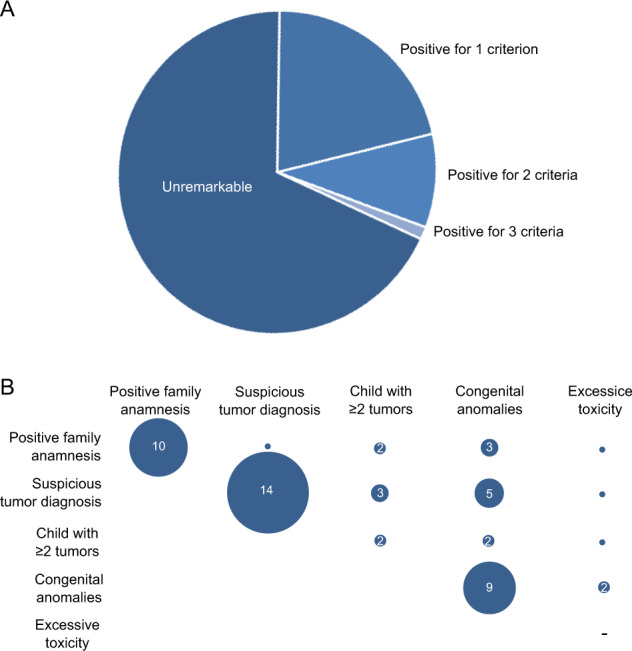
Overview of the patient´s clinical features. **A** Applying published criteria [5, 6], 51/160 patients were classified as being positive for at least one clinical criteria indicating a cancer predisposition. **B** This plot depicts how often a clinical criteria co-occurs with another clinical criteria in a given patient. The co-occurrence is indicated by the numbers in the circles, the smallest circles without a number represent single cases. Of note, the patients with excessive toxicity always harbor in addition another clinical sign. Hence, there are no patients who just harbor solely excessive toxicity as a single clinical sign (indicated by a “-ˮ). A suspicious tumor diagnosis occurred in 14 patients as the sole clinical sign whereas congenital anomalies often co-occur with a positive family anamnesis or a suspicious tumor diagnosis.

**Fig. 3:**
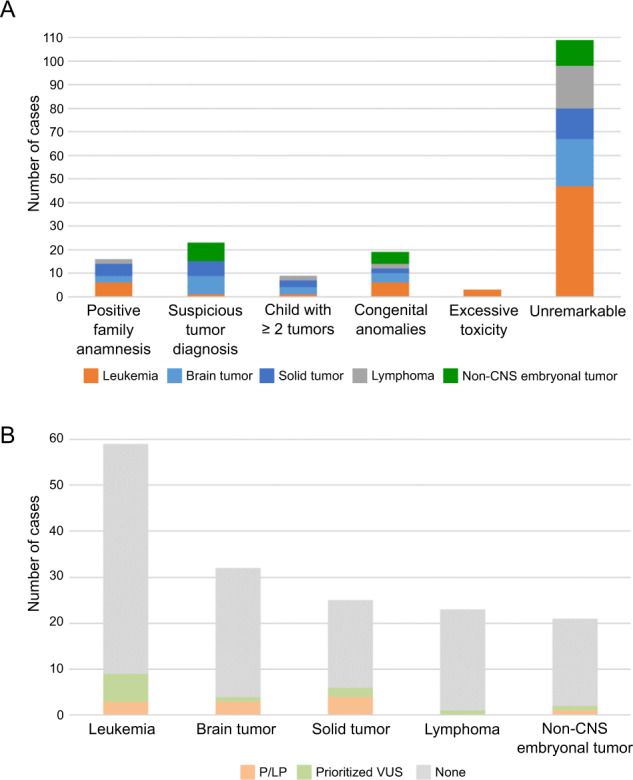
Phenotype-genotype correlation of the pediatric cancer patients. **A** The bar plot depicts the number of patients with or without clinical signs in relation to the tumor entity. **B** The bar plot depicts the number of patients with a certain type of cancer in relation to the presence of a pathogenic/likely pathogenic (P/LP) variant or prioritized variant of unknown significance (VUS).

To select patients with clinical suspicion for CPS, we applied published clinical selection criteria to our unselected cohort, which encompass family anamnesis (familial cancer history, consanguinity of parents), suspicious tumor diagnosis, child with multiple tumors, excessive toxicity during therapy, and preexisting congenital anomalies [5, 6]. Using this approach, we identified 51/160 patients (31.9%) who met at least one criterion (Fig. 2A). The majority of patients were positive for only one criterion (34/51 patients), while 15 patients were positive for two criteria, of which the majority had a congenital anomaly together with one other clinical criterion (10/15 patients) (Fig. 2A, B). Moreover, two patients were positive for three criteria suggesting an increased likelihood of a genetic cancer predisposition (Supplementary Table 1). The most frequent positive criterion was “suspicious tumor diagnosis” (23/51 patients), followed by patients with congenital anomalies (19/51 patients) and patients with a positive family history (16/51 patients) (Fig. 3A). Of note, we identified in the group of patients with leukemia a significant underrepresentation of patients with at least one positive criterion (OR: 0.41, *p* value: 0.02, Fisher’s exact test, Fig. 3A).

**Fig. 4:**
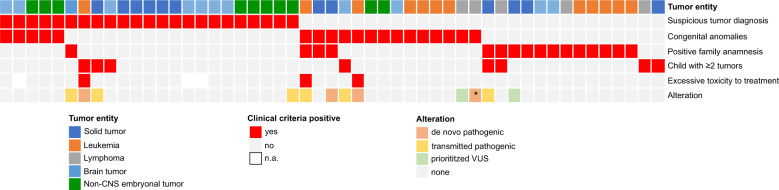
Summary of the phenotype-genotype correlation of the patients. Each line represents a clinical feature and each column represents a patient. Furthermore, we added whether a case is carrier of a P/LP alteration or a prioritized VUS. Asterisk (*) indicates that the patient harbored an imprinting disorder.

Next, we correlated the clinical data with the presence of pathogenic variants. Ten patients with P/LP variants in CPGs showed clinical signs indicating an underlying cancer predisposition syndrome (10/51, 19.6%), whereas one patient carrying a pathogenic *MSH6* variant was clinically unremarkable. Of the ten patients, two were positive for one criterion, six patients for two criteria, and two patients for three (Fig. 4). In contrast, for 80.4% of patients with positive clinical screening for an underlying CPS a genetic predisposition could not be identified. Interestingly, of the 11 patients carrying a prioritized VUS, ten harbored no clinical signs for an underlying cancer predisposition whereas one patient had a positive familial cancer history (Table 3, Supplementary Table 1).

**Table 3:** Overview of prioritized variants of unknown significance (VUS) potentially associated with cancer predisposition identified in the patient cohort.

Case; Diagnosis	Gene/transcript	Chromosomal position in bp (hg19)	Nucleotide change/ amino acid change	Zygosity	Inheritance	Clinical signs
Case-32; HL	*NBN* ENST00000265433.3	NC_000008.10:g.90983442_90983446del	c.657_661del p.(Lys219Asnfs*16)	heterozygous	Transmitted by mother	None
Case-37; B-ALL	*HOXB13* ENST00000290295.7	NC_000017.10:g.46805705C>T	c.251G>A p.(Gly84Glu)	heterozygous	Transmitted by father	None
Case-40; AML	*BRIP1* ENST00000259008.2	NC_000017.10:g.59761412_59761415del	c.2992_2995del p.(Lys998Glufs*60)	heterozygous	Transmitted by father	None
Case-52; OS	*CHEK2* ENST00000328354.6	NC_000022.10:g.29121228T>C	LRG_302t1:c.444+3A>G; Splicing	heterozygous	Transmitted by mother	1
Case-89; RMS	*CHEK2* ENST00000328354.6	NC_000022.10:g.29121087A>G	c.470T>C; p.(Ile157Thr)	heterozygous	Transmitted by father	None
Case-96; NB	*CHEK2* ENST00000328354.6	NC_000022.10:g.29091178C>A	c.1312G>T; p.(Asp438Tyr)	heterozygous	Transmitted by father	None
Case-102; B-ALL	*NBN* ENST00000265433.3	NC_000008.10:g.90990521T>C	c.511A>G p.(Ile171Val)	heterozygous	Transmitted by mother	None
Case-109; B-ALL	*CHEK2* ENST00000328354.6	NC_000022.10:g.29121087A>G	c.470T>C; p.(Ile157Thr)	heterozygous	Transmitted by mother	None
Case-132; MB	*FANCA* ENST00000389301.3	NC_000016.9:g.89831438G>A	c.2638C>T p.(Arg880*)	heterozygous	Transmitted by father	None
Case-142; B-ALL	*CHEK2* ENST00000328354.6	NC_000022.10:g.:29121230 C>T	LRG_302t1:c.444+1G>A; Splicing	heterozygous	Transmitted by father	None
Case-146; B-ALL	*CHEK2* ENST00000328354.6	NC_000022.10:g.chr22:29099499del	c.902del; p.(Leu301Trpfs*3)	heterozygous	Transmitted by father	None

*Diagnosis* diagnosis of initial cancer disease, *AML* acute myeloid leukemia, *B-ALL* B-cell acute lymphoblastic leukemia, *HL* Hodgkin lymphoma, *MB* medulloblastoma, *NB* neuroblastoma, *OS* osteosarcoma, *RMS* rhabdomyosarcoma, *Clinical signs* number of clinical signs indicating a cancer predisposition.

### Causative pathogenic variants in cancer predisposition genes

We have identified P/LP de novo variants associated with a CPS in four patients of which three patients were positive for at least one of the clinical criteria for cancer predisposition but none had a positive familial cancer history (Table 2).

We identified in Case-68 (B-ALL, 2.5 yrs) a de novo *PTPN11* variant (p.(Asn308Asp)). This variant was reported as pathogenic in associated with Noonan syndrome [17] and its deleterious effect on the protein functions has been described in several in vitro studies [18]. Hence, we established the diagnosis of a Noonan syndrome and concluded that the de novo *PTPN11* variant predisposed for the leukemia onset in Case-68. Case-140 (rhabdomyosarcoma, 4.6 yrs) carried a de novo LP splice region variant (LRG_214t2:c.6819+3del) in *NF1*. A neurofibromatosis (NF) type I patient has been reported in the LOVD database to carry a pathogenic base substitution at the same position where we detected the 1bp-deletion [19]. In line with the clinical presentation of the patient we established the diagnosis of NF type I for this patient. Moreover, we identified a de novo stopgain variant in *TP53* (p.(Arg196*)) in Case-99 which is described in ClinVar to be pathogenic in association with Li-Fraumeni Syndrome (LFS). Interestingly, the variant allele frequency of the variant was 17.2% based on WES (coverage of nucleotide 58x), indicating mosaicism, which was verified by PCR-based Sanger sequencing in the peripheral blood and the saliva of the patient. Despite the mosaic allele state of the *TP53* variant, the patient showed typical clinical features of a LFS patient (Supplementary Table 1). In Case-76 (glioblastoma, 12 years) a heterozygous, de novo 1 bp insertion within *MSH6*, leading to a frameshift and a preterminal stopcodon (p.(Gly1105Trpfs*3)) was detected. The in ClinVar as pathogenic described variant has been described in a family with Lynch syndrome [20]. Supporting our finding, immunohistochemistry of the tumor demonstrated loss of MSH2 and MSH6 protein expression. Hence, although no CPS clinical screening criteria were fulfilled we established the diagnosis of a Lynch syndrome due to the de novo *MSH6* variant.

In addition, we identified P/LP variants either maternally or paternally transmitted in five patients who all showed clinical signs of an underlying CPS (Supplementary Table 1). Three patients carried pathogenic *TP53* variants associated with a LFS (Table 2). In all three, the heterozygous *TP53* variants were inherited from the father. However, only in Case-19 and Case-31 a positive cancer history was reported (in both cases, a paternal uncle had a tumor during childhood). Notably, none of the fathers, aged 33–39 years at the time point of diagnosis of their children, were aware that they were carriers of a pathogenic cancer predisposing *TP53* variant. In addition, we identified pathogenic variants on which we have already reported in detail [13, 15], in *DICER1* in Case-10 diagnosed with DICER1 syndrome and in *MSH6* in Case-7 who has a constitutional mismatch repair deficiency.

### Prioritized variants of uncertain significance in genes known to predispose for adult-onset cancer

Next, by combination of the comprehensive clinical data of each patient with published data on VUSs, we identified six prioritized VUSs in *CHEK2* and *HOXB13* genes in seven patients for which we found strong evidence to confer an increased susceptibility to childhood cancer (Table 3). Of note, germline variants within those genes have been described to confer increased susceptibility to adult-onset cancer.

Case-52 (osteosarcoma, 11 yrs) showing no clinical features indicative of a CPS carried a maternally transmitted splice region variant in *CHEK2* (LRG_302t1:c.444+3A>G). Due to the lack of functional data on the impact of the splice variant, it is described as a VUS in association with breast cancer in ClinVar. Modeling of the variant shows decreases stability of the RNA duplex between the 5′ end of U1 snRNA and this splice donor (HBond score ∆1.9). Even if it does not impair the consensus sequence, based on the difference in the HBond score, it most likely leads to aberrant splicing [21] and, hence, to loss of its functional C-terminal kinase domain. Interestingly, the mother had breast cancer at the age of 45 years and the maternal grandmother pancreatic cancer (age of disease onset unknown). Several studies have reported an association between *CHEK2* variants and an increased risk, though with low-penetrance, for breast cancer [22]. Hence, we suggest that this splice variant predisposes to cancerogenesis in that family. Moreover, we identified four different prioritized VUSs in *CHEK2* in five additional cases of our cohort for which we suggest a role in cancer predisposition (Table 3). One of those CHEK2 variants is p.(Ile157Thr), which has been described to confer increased cancer risk for solid tumors and chronic lymphocytic leukemia [23, 24].

Case-37 (B-ALL, 3 yrs) carries a paternal transmitted p.(Gly84Glu) missense variant in *HOXB13*. None of the clinical signs indicative of a CPS were positive whereby the paternal grandfather had a colon carcinoma at the age of 55 years. Interestingly, carriers of the p.Gly84Glu variant have been described to have a significantly increased risk for solid tumors including colorectal cancer [25].

Taken together, the seven prioritized VUSs in *CHEK2* and *HOXB13* genes might confer an increased risk for childhood cancer. Notably, only one of those patients showed clinical signs indicating an underlying CPS.

### Prioritized variants of uncertain significance in genes associated with autosomal recessive cancer predisposing disorders

In addition, we identified prioritized VUSs in genes described to be associated with autosomal recessive disorders (Table 3). Although the variants were monoallelic and, thus, not syndrome-causing, we found strong evidence that they might lead to increased cancer susceptibility in the respective patients.

We identified pathogenic, heterozygous germline variants within the *NBN* gene in two patients (Cases −32 and −102). Homozygous or compound heterozygous variants within the *NBN* gene are associated with the autosomal recessive Nijmegen breakage syndrome (NBS) whereby an increased cancer susceptibility for heterozygous carriers of certain *NBN* variants has been reported [26]. In line, the truncating, heterozygous c.(657del5) *NBN* variant in Case-32 has been described as the Slavic founder variant occurring in >90% of NBS patients, and heterozygous carriers have an elevated cancer risk [27]. Case-102 (B-ALL, 17 yrs) carried a heterozygous maternally transmitted p.(I171V) variant. Heterozygous carriers of the p.Ile171Val have a significantly increased risk of developing ALL [28]. Hence, although both patients do not show clinical features of a NBS, the *NBN* variants might confer increased cancer susceptibility.

In addition, we identified two heterozygous truncating variants in members of the Fanconi anemia (FA) pathway, which were both transmitted by the respective fathers: p.(Lys998Glufs*60) in *BRIP1* (Case-40, AML) and p.Arg880* in *FANCA* (Case-132, medulloblastoma). Both variants are reported with low frequency in the gnomAD non-cancer population (MAF < 0.00002) and the *BRIP1* variant has been described as pathogenic in ClinVar. Heterozygous variants in genes of the FA pathway, including *BRIP1* and *FANCA*, have been described to predispose to breast cancer [29, 30].

### Potential role of digenic inherited variants in cancer predisposition

We have included in our study siblings, Case-77 and Case-78, who developed a pilocytic astrocytoma at the age of ten years and a Hodgkin lymphoma at the age of 18 years, respectively. Beside the fact that both siblings developed tumors, all clinical features of the individuals were unremarkable and the family cancer history was negative. Since both sisters developed tumors at a young age, we assume that a shared genetic aberration predisposes to cancer development in the siblings. We detected six shared variants in genes of category 1–3 of which none were classified as P/LP (Supplementary Table 3). Analysis of those shared variants for potential digenic variant combinations (refer to Supplementary Methods), we identified a high gene pair pathogenicity classification score between the variants in the DNA repair genes *RAD51C* and *NBN* (score 0.77), which are predicted to be two monogenic variants causing two different monogenic diseases. Notably, the maternally transmitted *NBN* and the paternally transmitted *RAD51C* variants are rare in the gnomAD non-cancer population (MAF: 0.00003 and 0.005, respectively). The *NBN* variant p.(Leu739Val) lies within a highly conserved domain that is crucial for the recruitment of ATM to DNA double-strand breaks [31]. The functional impact of the *RAD51C* variant p.(Thr287Ala) is unknown but it has been described to occur in breast and ovarian cancer patients as well as in the healthy population [32, 33]. Hence, we propose that a digenic variant combination, as for example *RAD51C* and *NBN*, which needs further functional evaluation, could play a role in the tumorigenesis in these siblings. In line, we already demonstrated for two patients (Case-1, Case-4) that functional complementation of digenic inherited variants takes indeed place [14, 16].

## Discussion

Genetic testing and the investigation of underlying CPS in pediatric oncology are of great interest to the affected families, given the high participation acceptance rate of 90% (Supplementary Fig. 1). In addition, knowledge of the presence of variants in CPGs that are involved in the DNA repair machinery, including mismatch and double-strand break repair associated CPS, can have direct implications for clinical decisions and personalized treatment options.

We present here a prospective study of 160 non-pre-selected patients with a complete set of clinical records, including detailed familial cancer history, on which family-based-trio sequencing has been performed. Despite the fact that we analyzed a clinically unselected cohort, we could observe a monogenetic causative variant in 6.9% of the patients with a P/LP cancer predisposing germline variant and in another 6.9% with a highly suspicious prioritized VUS, summing up to 13.8%. This is in line with recent publications that report pathogenic germline variants in 7–14% of children with cancer. The highest number of P/LP variants occurred in the patients with solid tumors (4/11 patients), followed by those with brain tumors and leukemia (each 3/11), which is in line with published findings [1].

Strikingly, we found that 31.9% of children in our unselected cohort showed at least one clinical finding suggestive of an underlying CPS. Of those clinically suspicious children, only 19.6% (10/51) carried a pathogenic alteration in a CPG (Fig. 4), consistent with recent publications reporting a frequency of 20–50% [3]. Vice versa, in 80.4% of cases with univocal clinical signs of an underlying genetic cancer predisposition, no genetic risk variant in a CPG was identified applying WES. Apart from the limitations of bioinformatics analysis workflows, the failure to identify a genetic cause might be attributed to different aspects: (i) The interpretation of variants is constrained by the knowledge of CPGs and their functions in CPS. Hence, most studies focused their analysis on only ~160 known CPGs [1–4], although in most of the cases, WES has been performed. In line, with this, although we expanded our set of analyzed genes (categories 2–3), we solely identified P/LP variants in category 1 genes that overlap with the known CPGs. (ii) The main focus has been on the identification of monogenetic predisposing variants whereas CPS might be also caused by potential digenic/oligogenic variant combinations. We propose this scenario as an underlying genetic mechanism in the siblings studied herein (Case-77/Case-78) in whom we detected VUSs in the DNA repair genes *NBN* and *RAD51C*, which might jointly lead to cancer susceptibility. (iii) Applying WES, only 2% of the genome and, hence, only a small spectrum of genetic alterations are analyzed. Especially, large structural variants are missed by this approach, although they have been identified as a cause of genetic cancer predisposition and somatic cancer diseases [34]. Hence, the application of new techniques, as whole-genome optical mapping or long read whole-genome sequencing, which are able to detect those genetic alterations, will likely contribute to our understanding of the genetic landscape in the future. (iv) Current clinical criteria are still not precise enough to detect only those patients who truly harbor a genetic alteration predisposing to cancer in childhood. On the other hand, applying more constrained criteria will undoubtedly result in increased numbers of patients who escape clinical attention.

Taken together, we present here a comprehensive WES analysis of a cohort of 160 unselected patients with childhood cancer and their parents. Both, pathogenic germline variants and prioritized VUSs, were detected in 6.9% of our patients, totaling up to 13.8% of the cohort with a monogenetic explanation of cancer onset. The systematic comparison of our WES-trio-data to clinical information revealed the complexity and challenges of establishing a genotype-phenotype correlation and emphasized the need for application of both study methods on the pediatric cancer population. Acknowledging the limitations of WES studies, additional molecular genetic methods including those that allow the detection of structural variations are required in order to investigate the full spectrum of germline predisposition in childhood cancer.

## Supplementary information

Supplementary Methods + Figures 1-2

Supplementary Tables 1-4
