# Supplementary material for: Comprehensive germline-genomic and clinical profiling in 160 unselected children and adolescents with cancer
Source: Eur J Hum Genet. 2021 Apr 12;29(8):1301–11. doi: 10.1038/s41431-021-00878-x (PMC8385053; doi:10.1038/s41431-021-00878-x)
Supplement: Supplementary file 1 — Supplementary Methods + Figures 1-2 [file 41431_2021_878_MOESM1_ESM.docx]

**Supplementary Methods**

**Bioinformatic analysis of whole-exome sequencing data**

*Variant calling*

Reads were extracted using bcl2Fastq v2.19.0 and adapters were trimmed using trimmomatic v0.33 (1). Afterwards, duplicate reads were marked using Picard tools 2.0.1 (http://broadinstitute.github.io/picard/). Alignment to human reference genome GRCh37.p7 (Ensembl Release 67, referring to GenBank assembly GCA_000001405.8) was done using BWA-MEM v0.7.12 (2) and Samtools v1.2 (3). To evaluate the coverage of the WES data, we applied bedtools v2.16.2 (4) and bamstats04 (http://dx.doi.org/10.6084/m9.figshare.1425030). We confirmed the familial relationship of the trio-WES data applying Peddy 0.4.6 (5). Single nucleotide variants (SNVs) and insertion/deletions (indels) were called using GATK v4.1.4.1 (filter settings for SNVs: QD<2.0, QUAL><0.0, SOR>2.25, MQ <40.0 and for indels: QD<2.0, QUAL<30.0, SOR>2.75, MQ <55.0) and VarScan2 v2.3.9 (6) (filter settings: min-coverage 10, min-var-freq 0.20, p-value 0.05, adj-var-freq 0.05, adj-p-value 0.15). Both variant callers were applied in the trio mode, allowing for differentiation between transmitted and *de novo* variants. In addition, platypus v0.8.1 (7) was used to call indels (default filter setting). All resulting SNVs from GATK and VarScan2 were combined into one SNV dataset whereby indels called by at least 2 of the 3 callers (GATK, VarScan2, Platypus) and all additional unique indels from GATK were considered for further analysis. Taken together, our final variant data set comprised of single nucleotide variants and small insertions/deletions.

*Variant filtering*

We excluded variants (i) with a variant allele frequency of <10% in the child, (ii) with a reported minor allele frequency (MAF) of >1% according to the gnomAD non-cancer population (8) and (iii) which occurred in ≥5% of cases in our cohort. The list of variants was further reduced to 295 genes, which can be separated into three different categories. Category one encompassed 151 genes described by Zhang *et* al. to be either autosomal dominant or autosomal recessive inherited, and to be tumor suppressor genes (9). In addition, genes described to be associated with rasopathies were included (10). Category two encompassed 114 genes not included in category 1 but (i) which were described by different germline studies to be associated with cancer predisposition (11–13), as well as (ii) different kinase genes described to be associated with cancer predisposition (9). Category three included 30 genes which have been described with germline variants most likely predisposing to certain tumors and which were not already included in group 1 and 2. Refer to table S2 for a complete overview of the genes analyzed.

*Variant annotation*

Functional annotation of variants was done using Ensembl Variant Effect Predictor v98.3(14). For *in silico* prediction of the effect of the variants, SIFT, Polyphen and CADD were applied. The COSMIC database (downloaded 25.03.2019 https.//cancer.sanger.ac.uk/cosmic/download) was used to identify variants located in somatic mutational hotspots. In addition, we used the ClinVar database (download 02/12/2019), the IARC *TP53* germline database and the LOVD database for *MSH2*, *MSH6*, *APC* and *NF1* in order to identify previously reported pathogenic variants. In addition, we used the dbNSFP 3.5 plugin to annotate the conservation scores based on GERP++ and phastCons100way_vertebrate. For *in silico* prediction of the effect of splice site variants, we applied the dbscSNV v1.1 plugin for VEP (15) which annotated the ada- and rf-scores to the splice variants. Furthermore, we applied the Human Splicing Finder 3.1 to classify the splicing effect of splice region variants (+/-3-8 bp).

**Analysis of digenic inheritance**

For analysis of potential digenic variant combinations in the siblings pair Case-77 and Case-78 we applied the online tool ORVAL (16). Here, we uploaded the 6 shared variants of the siblings in genes of category 1-3 to the platform. Using the results of the digenic combinations, we checked if the variants were truly digenic i.e. with one variant being either transmitted by mother and the other by the father or one variant being transmitted by one of the parents and the other being *de novo*. Using, as suggested by the tool, a pathogenicity classification score (CS) cut-off of >0.55 to identify high confidence digenic combinations, we ended up with one truly digenic combination in the sibling pair: *NBN* and *RAD51C* (CS:0.77).

**References**

1. Bolger AM, Lohse M, Usadel B. Trimmomatic: a flexible trimmer for Illumina sequence data. Bioinformatics. 2014;**30**:2114–20.

2. Li H, Durbin R. Fast and accurate short read alignment with Burrows-Wheeler transform. Bioinformatics. 2009;**25**:1754–60.

3. Li H, Handsaker B, Wysoker A, Fennell T, Ruan J, Homer N, et al. The Sequence Alignment/Map format and SAMtools. Bioinformatics. 2009;**25**:2078–9.

4. Quinlan AR, Hall IM. BEDTools: a flexible suite of utilities for comparing genomic features. Bioinformatics. 2010;**26**:841–2.

5. Pedersen BS, Quinlan AR. Who’s Who? Detecting and Resolving Sample Anomalies in Human DNA Sequencing Studies with Peddy. Am J Hum Genet. 2017;**100**:406–13.

6. Koboldt DC, Zhang Q, Larson DE, Shen D, McLellan MD, Lin L, et al. VarScan 2: somatic mutation and copy number alteration discovery in cancer by exome sequencing. Genome Res. 2012;**22**:568–76.

7. Rimmer A, Phan H, Mathieson I, Iqbal Z, Twigg SRF, WGS500 Consortium, et al. Integrating mapping-, assembly- and haplotype-based approaches for calling variants in clinical sequencing applications. Nat Genet. 2014;**46**:912–8.

8. Karczewski KJ, Francioli LC, Tiao G, Cummings BB, Alföldi J, Wang Q, et al. The mutational constraint spectrum quantified from variation in 141,456 humans. Nature. 2020;**581**:434–43.

9. Zhang J, Walsh MF, Wu G, Edmonson MN, Gruber TA, Easton J, et al. Germline Mutations in Predisposition Genes in Pediatric Cancer. N Engl J Med. 2015;**373**:2336–46.

10. Kuhlen M, Borkhardt A. Trio sequencing in pediatric cancer and clinical implications. EMBO Mol Med. 2018;**1**0.

11. Gröbner SN, Worst BC, Weischenfeldt J, Buchhalter I, Kleinheinz K, Rudneva VA, et al. The landscape of genomic alterations across childhood cancers. Nature. 2018;**555**:321–7.

12. Diets IJ, Waanders E, Ligtenberg MJ, van Bladel DAG, Kamping EJ, Hoogerbrugge PM, et al. High Yield of Pathogenic Germline Mutations Causative or Likely Causative of the Cancer Phenotype in Selected Children with Cancer. Clin Cancer Res. 2018;**24**:1594–603.

13. Huang K-L, Mashl RJ, Wu Y, Ritter DI, Wang J, Oh C, et al. Pathogenic Germline Variants in 10,389 Adult Cancers. Cell. 2018;**173**:355-370.e14.

14. McLaren W, Gil L, Hunt SE, Riat HS, Ritchie GRS, Thormann A, et al. The Ensembl Variant Effect Predictor. Genome Biol. 2016;**17**:122.

15. Jian X, Boerwinkle E, Liu X. In silico prediction of splice-altering single nucleotide variants in the human genome. Nucleic Acids Res. 2014;**42**:13534–44.

16. Renaux A, Papadimitriou S, Versbraegen N, Nachtegael C, Boutry S, Nowé A, et al. ORVAL: a novel platform for the prediction and exploration of disease-causing oligogenic variant combinations. Nucleic Acids Res. 2019;**47**:W93–8.

17. Richards S, Aziz N, Bale S, Bick D, Das S, Gastier-Foster J, et al. Standards and guidelines for the interpretation of sequence variants: a joint consensus recommendation of the American College of Medical Genetics and Genomics and the Association for Molecular Pathology. Genet Med. 2015;**17**:405–24.


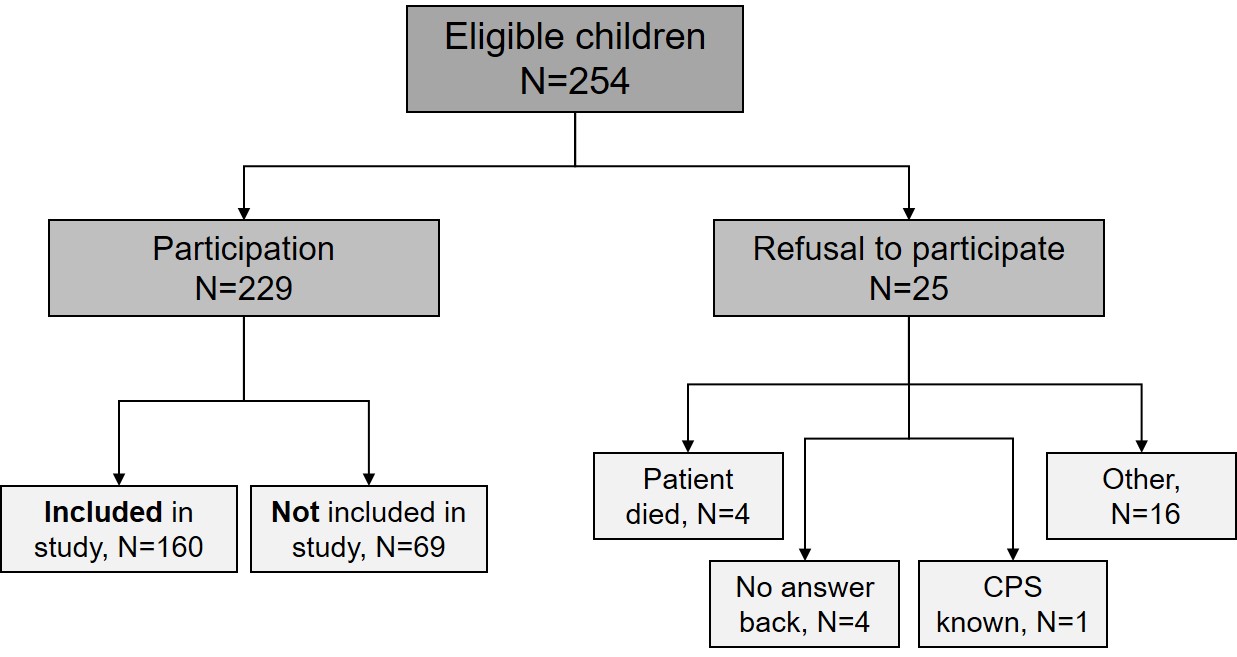


**Supplementary Figure 1:** Overview of the selected cohort. Overall 254 children diagnosed with cancer (01/2015-04/2019) were asked to participate in our study. Of these 229 patients and their parents gave their consent to participate, yielding a participation rate of 90.2%. Of these, 160 patients and their parents were included in the study, while 69 patients were not included due to missing material of one of the trio or due to quality problems (material or sequencing data). In total 25 patients and their parents refused to participate in this study due to different reasons: 4 patients died before they were included in the study; 4 families did not give a final answer-back; for one patient the diagnosis of a cancer predisposition syndrome was already established prior to cancerogenesis and 16 families gave no consent to participate due to cultural reasons or out of fear.


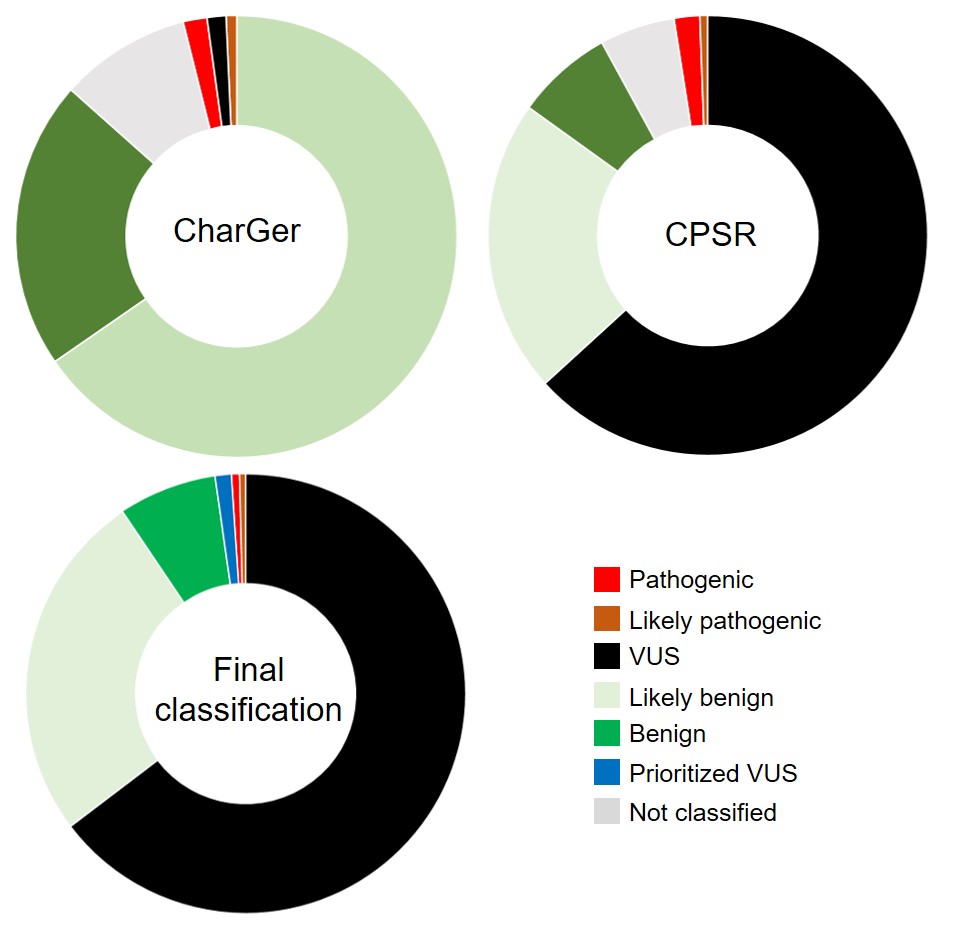


**Supplementary Figure 2:** Overview of the identified variants in genes of category 1 classified according to the American College of Medical genetics (ACMG) guidelines (17) using the automated tools CharGer and CPSR, as well as the final classification after manual revision of the variants. Only a small number of the variants were classified as pathogenic and likely pathogenic variants or prioritized variants of unknown significance (VUSs) in the final classification (2.2%, 20/899 variants).
